# Photodynamic Diagnosis-Assisted Transurethral Resection Using Oral 5-Aminolevulinic Acid Decreases the Risk of Repeated Recurrence in Non-Muscle-Invasive Bladder Cancer: A Cumulative Incidence Analysis by the Person-Time Method

**DOI:** 10.3390/diagnostics11020185

**Published:** 2021-01-28

**Authors:** Makito Miyake, Nobutaka Nishimura, Yasushi Nakai, Tomomi Fujii, Takuya Owari, Shunta Hori, Yosuke Morizawa, Daisuke Gotoh, Satoshi Anai, Kazumasa Torimoto, Nobumichi Tanaka, Yoshihiko Hirao, Kiyohide Fujimoto

**Affiliations:** 1 Department of Urology, Nara Medical University, Kashihara, Nara 634-8522, Japan; ffxxxx.nqou@gmail.com (N.N.); nakaiyasusiuro@live.jp (Y.N.); tintherye@gmail.com (T.O.); horimaus@gmail.com (S.H.); tigers.yosuke@gmail.com (Y.M.); dgotou@gmail.com (D.G.); sanai@naramed-u.ac.jp (S.A.); torimoto@naramed-u.ac.jp (K.T.); 2 Department of Diagnostic Pathology, Nara Medical University, Kashihara, Nara 634-8522, Japan; fujiit@naramed-u.ac.jp; 3 Department of Prostate Brachytherapy Nara Medical University, Kashihara, Nara 634-8522, Japan; sendo@naramed-u.ac.jp; 4 Department of Urology, Osaka Gyoumeikan Hospital, Konohana-ku, Osaka 554-0012, Japan; hiraoyos@gmail.com

**Keywords:** bladder cancer, urothelial carcinoma, transurethral resection, fluorescence, photodynamic diagnosis, 5-aminolevulinic acid, repeated recurrence, prognosis, person-time method

## Abstract

Clinical evidence regarding risk reduction of repeated bladder recurrence after initial photodynamic diagnosis (PDD)-assisted transurethral resection of bladder tumor (TURBT) is still limited in patients with non-muscle-invasive bladder cancer (NMIBC). We analyzed patients with primary NMIBC undergoing TURBT without any adjuvant treatment to compare the risk of cumulative recurrence between the conventional white-light (WL)-TURBT and PDD-TURBT. Out of 430 patients diagnosed with primary NMIBC from 2010 to 2019, 40 undergoing WL-TURBT and 60 undergoing PDD-TURBT were eligible. Multivariate Cox regression analysis for time to the first recurrence demonstrated that PDD assistance was an independent prognostic factor with better outcome (*p* = 0.038, hazard ratio = 0.39, and 95% confidence interval 0.16–0.95). While no patient experienced more than one recurrence within 1000 postoperative days in the PDD-TURBT group, five out of 40 patients treated by WL-TURBT experienced repeated recurrence. The comparison of cumulative incidence per 10,000 person-days between the two groups revealed that PDD assistance decreased by 6.6 recurrences per 10,000 person-days (exact *p* = 0.011; incidence rate ratio 0.37, Clopper–Pearson confidence interval 0.15–0.82). This is the first study addressing PDD assistance-induced risk reduction of repeated bladder recurrence using the person-time method. Our findings could support clinical decision making, especially on adjuvant therapy after TURBT.

## 1. Introduction

Transurethral resection of bladder tumor (TURBT) is the mainstay of treatment and diagnosis in bladder cancer, especially in non-muscle-invasive bladder cancer (NMIBC), accounting for about 80% of bladder cancers [1].

Although the bladder can be preserved in most patients with NMIBC, frequent and repeated intravesical recurrence after TURBT remains the biggest clinical problem. Our previous study demonstrated that smoothing of the recurrence hazard curve showed three peaks: a large first peak before 500 postoperative days, a small second peak at 500–1000 days, and another small peak around 1500 days [2]. When the former two peaks are defined by ‘early recurrence,’ it is considered that the early recurrence is largely attributed to tiny or flat lesions, which are easily overlooked by conventional white-light (WL)- TURBT [2,3].

Improving the quality of TURBT and increasing the rate of complete tumor resection is essential to reduce the risk of recurrence. Over the last couple of decades, 5-aminolevulinic acid (5-ALA) or hexaminolevulinate-induced fluorescence cystoscopy has been developed with the aim of detecting tiny and/or flat lesions [4,5]. Recently, the clear benefit of photodynamic diagnosis (PDD)-assisted TURBT using 5-ALA in terms of improved tumor detection rate and better oncological outcome has been confirmed in Japan [6,7,8]. Based on favorable evidence, oral 5-ALA was approved as an intraoperative diagnostic drug in Japan in December 2017, and its use has been strongly recommended in the Japanese Urological Society-edited *Clinical Practice Guidelines for Bladder Cancer 2019* [9].

It has been almost three years since the approval of the PDD-TURBT technique in Japan. However, the clinical evidence regarding oncological outcomes is still limited, especially in repeated recurrence after initial TURBT. Here, we analyzed patients with primary NMIBC undergoing TURBT without any adjuvant treatment to compare the risk of cumulative recurrence between WL-TURBT and PDD-TURBT.

## 2. Materials and Methods

### 2.1. Patients and Data Collection

The Department of Urology, Nara Medical University, participated in investigator-initiated multicenter clinical trials (the AURORA and ALA-BC-1 phase II/III trials) from February 2012 to December 2012 and a sponsor-initiated multicenter clinical trial (the SPP2C101 phase III trial) from May 2015 to March 2016. Our hospital provided the PDD-TUBT technique for patients with NMIBC as a framework of an advanced medical care system, namely ‘ALAB-O,’ from July 2010 to December 2013. The results have adequately proven the efficacy and safety of PDD-TURBT using oral 5-ALA in patients with NMIBC. Since the Pharmaceuticals and Medical Devices Agency (PMDA) approval in Japan (December 2017), we have started the PDD-TUBT for NMIBC as a medical practice covered by regular health insurance.

We reviewed 430 patients with primary NMIBC undergoing WL-TURBT or PDD-TURBT from July 2010 to August 2019 at the Department of Urology, Nara Medical University hospital. The clinical characteristics of the patients included age, sex, performance status, past history of NMIBC, tumor multiplicity, tumor size, and second transurethral resection. Pathological diagnosis of the specimens was reassessed by an experienced uropathologist (T.F.) to determine T categories (2009 Union for International Cancer Control TNM Staging System), tumor grades (2004 World Health Organization (WHO) classifications), presence of carcinoma in situ (CIS), and lymphovascular involvement.

### 2.2. Surgical Procedure and Device

The preoperative process for administration of 5-ALA, anesthesia, resection device, and imaging device for WL-TURBT and PDD-TURBT have been previously described [7,8,10]. Approximately 3 h (range, 2–4 h) before surgery, patients orally received a water-dissolved 5-ALA hydrochloride solution at a dose of 10 or 20 mg/kg. Although both doses of 5-ALA were clinically applicable, the latter is used in current clinical practice. PDD assistance during surgery was carried out with the Storz D-LIGHT System (KARL STORZ GmbH & Co. KG; Tuttlingen, Germany) (February 2012– March 2016) or the Storz Professional Image Enhancement System (IMAGE1 S^TM^, KARL STORZ GmbH & Co. KG; Tuttlingen, Germany) (January 2018–August 2019). We switched imaging modes of white light and blue light frequently during the operation to set the resection margin. Resection of all the visible tumors accompanied with or without cold cup biopsy was performed according to the surgical method previously described.

### 2.3. Follow-Up after Initial TURBT

Basically, a second TUR was indicated if high-grade Ta or T1 was pathological detected or there was no adequate muscularis propria in the specimen. Whether or not a second TUR was performed depended on the judgment of the physician. Single immediate instillation of chemotherapeutic drugs (epirubicin or pirarubicin) and adjuvant therapy depended on the physician’s preference. Patients were followed up according to our institutional protocol as previously described. Briefly, WL cystoscopy with urine cytology was performed every three months for two years, every six months for the subsequent three years, and annually thereafter. Recurrence in the pelvis and upper urinary tract was checked using computed tomography every six or twelve months. Recurrence was defined as recurrent intravesical tumors of pathologically proven urothelial carcinoma (UC). Progression was defined as recurrent disease with the invasion of the muscularis propria (≥T2), positive regional lymph nodes, and/or distant metastases.

### 2.4. Statistical Analysis

Prism software version 7.00 (GraphPad Software, Inc., San Diego, CA, USA) and SAS statistical software package version 9.4 (SAS Institute Inc., Cary, NC, USA) were used for data visualization and statistical analyses. In this study, *p*-values < 0.05 were considered statistically significant. After the patient selection, Fisher’s exact test or the Mann–Whitney *U* test was applied to compare the background between the WL-TURBT group and the PDD-TURBT group. The recurrence-free survival was estimated using Kaplan–Meier analysis and compared using the log-rank test or univariate Cox regression analysis between the two groups. Multivariate Cox proportional hazards regression analysis was used to identify independent prognostic variables. Variables that potentially affected prognosis on univariate analysis or that presented significant differences during the background comparison were included in the multivariate analysis.

Incidence rates of bladder recurrence after initial TURBT were calculated by dividing the number of cumulative incidences by the total number of person-days at risk throughout the follow-up period. The incidence rates were compared between the WL-TURBT group and the PDD-TURBT group by calculating the normal approximation confidence interval, incidence rate ratio (Clopper–Pearson confidence interval), and exact *p*-value under conditioning on the total number of cases, which follows a Poisson distribution [11,12,13].

## 3. Results

**Figure 1:**
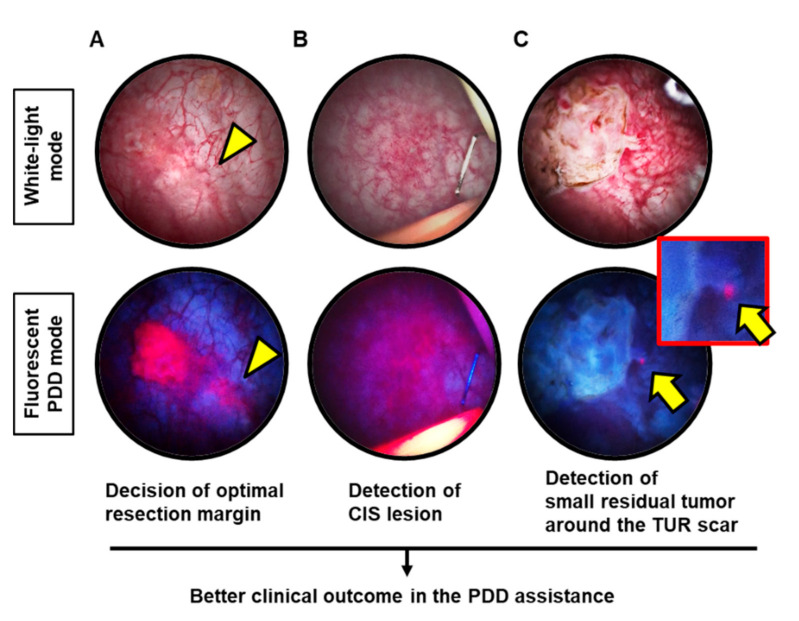
Representative images showing the benefit of using PDD assistance during TURBT. (**A**) A papillary Ta low-grade tumor is examined by both white-light (WL) and photodynamic diagnosis (PDD) modes. A small lesion (yellow arrowhead) spreads in the skirt of the tumor, which is clearly identified by PDD. (**B**) This lesion is equivocal on WL mode, while the PDD mode reveals a flat tumor fluorescing pink under PDD mode. The pathological examination of the biopsy confirmed that this lesion was carcinoma in situ. (**C**) After tumor resection, the PDD mode found a small residual tumor (yellow arrow) close to the resection margin. The additional resection was performed to achieve complete tumor resection. CIS: carcinoma in situ; TUR: transurethral resection.

We have accumulated experience with 5ALA-mediated PDD-TURBT since 2004. The visually stunning endoscopic technique can enable surgeons to determine circumferential resection margin and often detects occult tumors that are easy to overlook under observation with WL mode (Figure 1A,B). Bladder UC often grows with a skirt of tumor around the base. In addition, post-resection observation with PDD mode often captures small residual tumors (Figure 1C).

**Figure 2:**
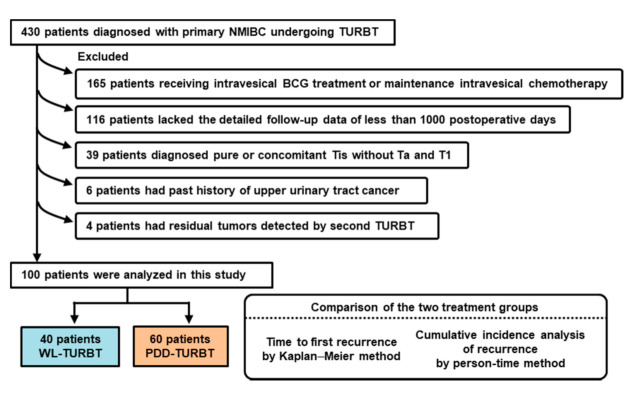
Study design and flow chart of this study. NMIBC: non-muscle-invasive bladder cancer; TURBT: transurethral resection of bladder tumor; BCG: Bacillus Calmette Guérin; WL: white-light; PDD: photodynamic diagnosis.

Figure 2 shows a flowchart of the patient selection process. The main purpose of this study was to evaluate the real benefit of PDD-TURBT through the sensitive tumor detection and subsequent complete tumor resection. Because any adjuvant treatment could hinder the evaluation, patients treated with adjuvant intravesical treatment of Bacillus Calmette Guérin (BCG)/chemotherapy were excluded from the analysis. Further, patients treated with adjuvant intravesical treatment of BCG/chemotherapy, patients lacking detailed follow-up data of less than 1000 postoperative days, patients with pure Tis without Ta and T1, patients with concurrence/past history of upper urinary tract cancer, and patients with residual tumors detected by a second TURBT were excluded from 430 patients with primary NMIBC. A hundred patients (28%) were eligible for the analysis, in which tumors were considered to be completely resected and, therefore, did not require any adjuvant repeated intravesical treatment. Of 100 patients, 40 (40%) and 60 (60%) underwent WL-TURBT and PDD-TURBT, respectively. Table 1 shows the patients’ clinicopathologic variables and a comparison between the two groups. Although the population with tumor size ≥ 3 cm was higher in the WL-TURBT group (*p* = 0.04), there was no significant difference in other factors between the two groups.

**Figure 3:**
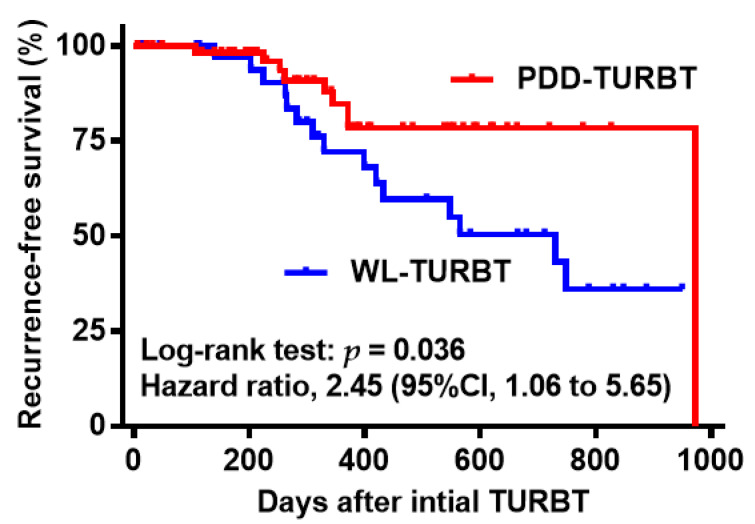
Recurrence-free survival curves of patients treated with white-light transurethral resection of bladder tumor (WL-TURBT) or photodynamic diagnosis (PDD)-assisted (TURBT).

The median follow-up period was 402 days (interquartile range, 212–688). Fifteen (38%) out of 40 patients treated by WL-TURBT and nine (15%) out of 60 patients treated by PDD-TURBT experienced at least one recurrence within 1000 days after initial TURBT. Recurrence-free survival was significantly longer in the PDD-TURBT group compared to the WL-TURBT group (Figure 3). Univariate Cox regression analysis revealed that only PDD-assisted surgery was significantly associated with better outcome (Table 2; *p* = 0.023, hazard ratio = 0.35) and that large tumor size was the most likely to be a confounding factor (*p* = 0.21; hazard ratio = 1.84) among other clinicopathologic variables. Multivariate analysis including other prognostic factors demonstrated that PDD-assisted surgery was an independent prognostic factor with better outcome (Table 2; *p* = 0.038, hazard ratio = 0.39). Among the 100 analyzed patients, only one patient had progression to muscle-invasive disease. The risks of progression and cancer-specific death were unable to be evaluated in this study.

**Figure 4:**
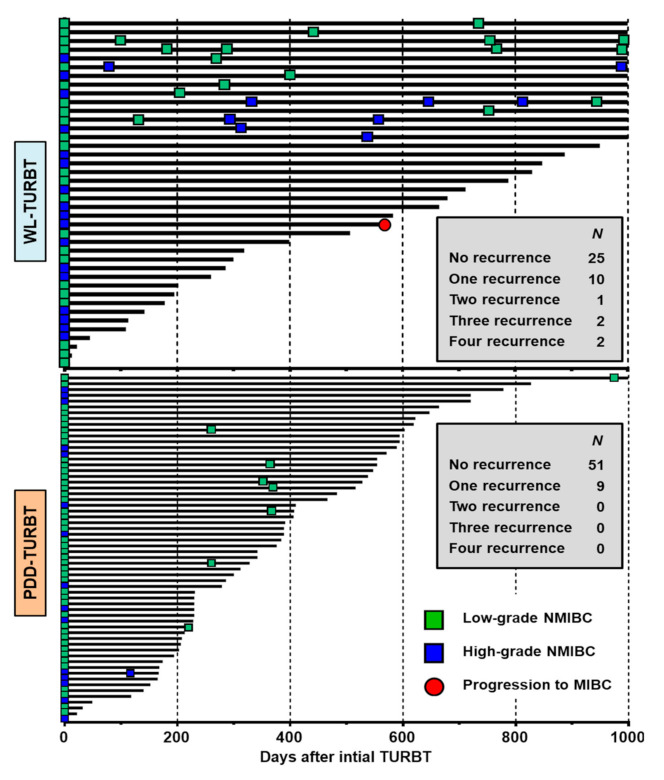
Swimmer’s plot of the 100 patients with time on follow-up. Individual swimmer’s plots for each patient showing the number of follow-up days for the WL-TURBT group (upper figure) and the PDD-TURBT group (lower figure). Follow-ups were performed by routine white-light cystoscopy and conventional urine cytology. Green and blue square boxes indicate low-grade and high-grade non-muscle-invasive bladder cancer (NMIBC), respectively. The red circle indicates a recurrent tumor of muscle-invasive bladder cancer (MIBC). The number of patients who experienced repeated recurrence, ranging from one to four, is shown in each figure.

While there was no patient who experienced more than one recurrence within 1000 postoperative days in the PDD-TURBT group, one (2.5%), two (5%), and two (5%) patients out of 40 patients treated by WL-TURBT experienced two, three, and four repeated recurrences within 1000 postoperative days. Figure 4 presents a swimmer’s plot of the 100 patients, with time on follow-up days by routine cystoscopy and urine cytology shown for each patient. The plot shows both each patient’s time of recurrence and pathological tumor grade of recurrent disease (low-grade NMIBC or high-grade NMIBC). Table 3 shows the comparison of cumulative incidence per 10,000 person-days between the two groups. The use of PDD assistance was associated with a decrease of 6.6 recurrences per 10,000 person-days (*p* = 0.011).

## 4. Discussion

Single event times are mostly analyzed using the Kaplan–Meier method followed by the log-rank test or the Cox proportional hazards model. However, patients can suffer from repeated same events in some types of disease, such as cardiovascular events [14], biliary colic due to gallbladder stone [15], urinary symptoms after radiotherapy in prostate cancer [16], and bladder recurrence of NMIBC [17,18]. Simon et al. reported that repeated (two or more) recurrences of TaG1 tumors in patients with primary TaG1 was not associated with disease progression to high-grade Ta/T1 or muscle-invasive disease but increased the risk of subsequent recurrences [18]. Thus, more effort should be made to investigate the natural history of repeated recurrence after initial treatment and to decrease the risk of repeated recurrence. Previous studies have applied the extended Cox proportional hazards mode or multiple-event and event-count models to account for repeated events [17,18]. However, these statistical methods are considered too complicated to apply to our clinical practice. In this study, we utilized the person-time method to compare the risk of repeated recurrence between conventional WL-TURBT and PDD-TURBT.

Our study demonstrated that the use of PDD assistance decreases not only the rate of the first recurrence but subsequent recurrences. The person-time method found a 63% reduction of recurrence (from 10.5 to 3.9 per 10,000 person-days). Regarding the associated medical cost, one recurrence requires approximately 4000 USD for hospitalization expenses, anesthesia, and TURBT in Japan. Moreover, psychological and physical stress is unfathomable in patients having recurrent tumors. Early recurrence within 1000 postoperative days is considered largely attributed to tiny lesions, which are overlooked by conventional WL TURBT [2,3]. PDD contributes to the detection of those tumors, leading to complete resection of multiple tumors. Another hypothesis of the PDD-induced risk reduction of repeated recurrence is potential photodynamic therapy (PDT) during PDD-TURBT. When the fluorescent substance of protoporphyrin IX (PPIX), which predominantly accumulated in tumor cells exposed to 5-ALA, is excited by red or green light irradiation, reactive oxygen is produced, which damages the tumor [19]. Laser irradiation, followed by the administration of photoreactive agents (not 5-ALA), has been the current trend of the PDT for bladder cancer [20]. During the 2000s, several clinical studies investigated the potential of 5-ALA-based PDT mainly for treatment-refractory NMIBC, including BCG-refractory CIS [19,21,22,23]. Among these studies, Waidelich et al. performed whole bladder PDT using 5-ALA and irradiation by 100 J/cm^2^ white-light source for patients with recurring, multifocal, Ta UC, or CIS and concluded that the procedure was clinically applicable [22]. At the moment, we have no available data regarding how much irradiation (J/cm^2^) is administered to tumor cells during PDD-TURBT. Further basic research is warranted to confirm our hypothesis regarding the potential of PDT during PDD-TURBT.

Our previous study developed a risk stratification model for NMIBC, so-called J-NICE (the Japanese NIshinihon uro-onCology Extensive collaboration group) risk tables [24]. The recurrence-specific table included six factors as follows: age, primary or recurrent, multiplicity, tumor size, T category, and tumor grade. In the univariate prognostic analysis of this study, none of these factors was associated with the risk of first recurrence (Table 2). According to the J-NICE score [24] and EORTC (the European Organisation for Research and Treatment of Cancer) risk score table [25], high tumor grade is associated with higher risk of bladder recurrence in comparison to low tumor grade. Table 2 describes the consistent result, which, however, did not reach statistical significance (hazard ratio = 1.14, 95% confidence interval, 0.14–9.5) largely due to low detection power and small sample size. A future study using a larger sample size is definitely required in order to evaluate the true relationship between tumor grade and clinical outcome in the PDD-TURBT era. However, PDD assistance during TURBT was identified as an independent prognostic factor in the multivariate analysis. Given that the main purpose of this study was to evaluate the capability of complete surgical resection of PDD-TURBT, only primary NMIBC patients who did not receive BCG therapy or maintenance intravesical chemotherapy were eligible for the analysis. This study was a single center-based retrospective investigation, therefore several potential biases might have affected the results. Another limitation of this study is that the sample size was relatively small, with only 40 patients in the WL-TURBT group and 60 patients in the PDD-TURBT.

## 5. Conclusions

To the best of our knowledge, this is the first study addressing the PDD assistance-induced risk reduction of repeated bladder recurrence using the person-time method. We believe that our findings could support clinical decision making, especially on adjuvant therapy after TURBT. Further large-scale retrospective studies using background adjustment method, such as propensity score matching, and subsequent prospective trials are needed in order to better define the real clinical value of PDD-TURBT.

## Figures and Tables

**Table 1:** Clinicopathologic variables of patients treated with WL-TURBT or PDD-TURBT.

Variables	Total	WL-TURBT	PDD-TURBT	*p* Value
N	100	40	60	
Age, mean ± *SD*	75.3 ± 9.0	73.9 ± 11.0	76.2 ± 7.3	0.26 #
Sex				0.79 ##
Male	89	36 (90%)	53 (88%)	
Female	11	4 (10%)	7 (12%)	
Multiplicity				0.46 ##
Single	48	21 (53%)	27 (45%)	
Multiple	52	19 (48%)	33 (55%)	
Tumor size				0.04 ##
Less than 1 cm	17	6 (15%)	11 (18%)	
1–3 cm	62	19 (48%)	43 (72%)	
3 cm or more	21	15 (37%)	6 (10%)	
T category				0.066 ##
Ta	68	23 (58%)	45 (75%)	
T1	32	17 (42%)	15 (25%)	
Tumor grade (WHO 2004)				0.13 ##
Low-grade	71	25 (63%)	46 (77%)	
High-grade	29	15 (37%)	14 (23%)	

WL-TURBT, white-light transurethral resection of bladder tumor; PDD-TURBT, photodynamic diagnosis-assisted transurethral resection of bladder tumor; SD, standard deviation; WHO, the World Health Organization; # Mann-Whitney U test; ## Fisher’s exact test.

**Table 2:** Prognostic variables for recurrence-free survival after TURBT.

Variables		Univariate Analysis			Multivariate Analysis		
		HR	95% CI	*p*-Value	HR	95% CI	*p*-Value
Type of TURBT	WL-TURBT	1			1		
	PDD-TURBT	0.35	0.15–0.87	0.023	0.35	0.14–0.92	0.032
Age (years)	Less than 70	1					
	70 or more	1.03	0.91–1.17	0.64			
Sex	Male	1					
	Female	0.89	0.19–4.07	0.88			
Multiplicity	Single	1					
	Multiple	1.04	0.96–1.12	0.34			
Tumor size	Less than 3 cm	1			1		
	3 cm or more	1.84	071–4.80	0.21	1.01	0.98–1.04	0.47
T category	Ta	1					
	T1	0.82	0.34–1.97	0.66	0.57	0.09–3.3	0.53
Tumor grade	Low-grade	1					
	High-grade	0.72	0.26–1.96	0.66	1.14	0.14–9.5	0.90

WL-TURBT: white-light transurethral resection of bladder tumor; PDD-TURBT: photodynamic diagnosis-assisted transurethral resection of bladder tumor; HR: hazard ratio; CI: confidence interval.

**Table 3:** A cumulative incidence analysis of bladder recurrence by person-time method.

Surgical Method	WL-TURBT	PDD-TURBT
Patients	40	60
Cumulative incidence (bladder recurrence)	26	9
Person-days at risk	24,855	23,174
Incidence (per 10,000 person-days)	10.5	3.9
Normal approximation confidence interval	6.44 to 14.5	1.35 to 6.42
Incidence rate ratio (Clopper–Pearson confidence interval)	-	0.37 (0.15 to 0.82)
Exact *p* value	-	0.011

WL-TURBT: white-light transurethral resection of bladder tumor; PDD-TURBT: photodynamic diagnosis-assisted transurethral resection of bladder tumor.

## Data Availability

The datasets used and/or analyzed during the current study are available from the corresponding author on reasonable request.
